# Current Trends of Human Adenovirus Types Among Hospitalized Children—A Systematic Review

**DOI:** 10.3390/v17070914

**Published:** 2025-06-27

**Authors:** Janina Soler Wenglein, Luca Scarsella, Christine Kotlewski, Albert Heim, Malik Aydin

**Affiliations:** 1 Department of Pediatrics, Protestant Hospital of the Bethel Foundation, Medical School and University Medical Center East Westphalia-Lippe, Bielefeld University, 33617 Bielefeld, Germany; janina.soler@uni-bielefeld.de (J.S.W.);; 2 Laboratory of Experimental Pediatric Pneumology and Allergology, Department of Human Medicine, Faculty of Health, Witten/Herdecke University, 58453 Witten, Germany; luca.scarsella@uni.wh.de; 3 Medical School OWL, Bielefeld University, 33615 Bielefeld, Germany; 4 Department of Anesthesiology, Center for Clinical and Translational Research, Helios University Hospital Wuppertal, Witten/Herdecke University, 42283 Wuppertal, Germany; 5 Institute of Virology, Hannover Medical School, Carl-Neuberg-Str. 1, 30625 Hannover, Germany; 6 Virology and Microbiology, Center for Biomedical Education and Research (ZBAF), Department of Human Medicine, Faculty of Health, Witten/Herdecke University, 58453 Witten, Germany; 7 Children’s Hospital, Vestische Kinder- und Jugendklinik Datteln, Witten/Herdecke University, 45711 Datteln, Germany

**Keywords:** human adenovirus, infection, respiratory tract, gastrointestinal tract, immunosuppression, children

## Abstract

Human adenoviruses (HAdVs) are pathogens causing different illnesses, particularly in pediatric and immunocompromised patients in developed countries. The clinical spectrum of HAdV-infections ranges from mild to severe, and the clinical presentation varies widely. Certain HAdVs types, including types B3, E4, B7, B14, B21, G55, and B66, may be associated with lower respiratory tract infections and thus lead to higher hospitalization, increased morbidity, as well as lethality rates. The aim of this article is to synthesize and analyze the prevalence of specific HAdV types in pediatric patients worldwide. A systematic literature search was performed using MEDLINE, Scopus, and Web of Science. In total, *n* = 1167 titles and abstracts were screened, and 105 full-text articles were assessed for eligibility. Screening, data extraction, and appraisal were analyzed by reviewers, in accordance with PRISMA guidelines and JBI recommendations. We included studies reporting on currently circulating HAdV types (*n* = 16). Based on a systematic and narrative approach, relevant types of HAdV biology and infections in children are presented. In detail, HAdV-B3 and HAdV-B7 were commonly associated with severe respiratory tract infections, while HAdV-F40 and HAdV-F41 caused acute gastroenteritis. Moreover, detailed research revealed the critical role of HAdV-C2 and the necessity for particular attention to HAdVs in acute neurological infections. This comprehensive analysis highlights the significant global distribution and diverse clinical implications of different HAdV types in children, pointing out the need for continued surveillance to better understand HAdVs epidemiology and its implications for public health, and future preventive measures, in particular among vulnerable patients.

## 1. Introduction

Human adenoviruses (HAdVs) are non-enveloped, double-stranded DNA viruses and relatively large in terms of size. HAdVs belong to the Mastadenovirus genus of the *Adenoviridae* family [1], classified into seven different species from A to G [2], and 116 different genotypes described yet [3,4]. They have a 95 nm pseudoicosahedral capsid composed mainly of different hexon, penton base, and fiber proteins. The hexon represents about 60% of the virion and is highly conserved across the *Adenoviridae* family. The penton complex at each vertex includes a penton base and a fiber, which aids in receptor interaction, and minor proteins (IIIa, VI, VIII, IX) may help to stabilize the capsid [5]. For cell entry, the Coxsackie-adenovirus receptor, CD46, and Desmoglein-2 (Dsg-2) are, among others, relevant HAdVs surface receptors. In addition, host soluble factors, including factor IX and complement protein C4BP may also enhance viral transduction by bridging adenoviral fibers to the cell membrane [6,7,8,9,10,11,12,13]. In a clinical context, HAdVs may cause a broad range of infections in several organ systems. Of note, there are significant regional and type-related differences in the seroprevalence of HAdVs. While some HAdV types, including HAdV-C2, have an increased global seroprevalence (36 to 92%), others, such as HAdV-D26, are significantly less common (≤12% in the USA and Europe) [14]. Among pediatric patients, HAdVs commonly affects children under 5 years of age, with infants under two years of age are at higher risk of prolonged hospitalization due to respiratory tract infections [15]. Furthermore, HAdVs infections are frequently observed between six months to five years of age, in particular in immunocompromised children [16], and several cases may occur from a reactivation of HAdVs latency [9]. Although eye, upper respiratory tract, and gastrointestinal infections caused by HAdVs may cause significant morbidity, lower respiratory tract infections (LRTIs) present an increased risk of severe diseases. They can be critical in immunocompetent, as well as immunocompromised children [17,18,19].

However, due to the heterogeneous presentation of HAdVs infections, especially in susceptible patient groups, a comprehensive understanding of HAdVs in pediatric patients is required for further preventive measures. Moreover, information and awareness regarding the clinical relevance of HAdVs are urgently necessary. Therefore, the major aim of this article is to describe the clinical presentation of emerging HAdV types, particularly in hospitalized children. To address this, our systematic review aims to overview HAdVs biology and pathophysiology, followed by a systematic literature search on newly reported HAdV types with their clinical presentation that will emphasize the relevance of HAdVs in clinical contexts. Consequently, a deeper understanding of this context may result in better diagnostic, therapeutic, and preventive measures.

### 1.1. The Molecular Role of HAdV in the Context of Virus Infection

HAdVs are ubiquitous pathogens found in both humans and animals and are highly stable in the environment [20,21]. There is no zoonotic transmission from animals to humans that has been described yet, but cross-species transmission between different animal species was already observed [22]. HAdVs may remain on any surface and can be stable at room temperature for several weeks, making it challenging to break chains of infections [23,24]. The respiratory route (e.g., through droplets and aerosols) is the most common way of transmission [25]. The second most common route of transmission is fecal–oral, occurring, for example, through contaminated food, water, or direct/close contact [25].

After entering the upper respiratory and gastrointestinal tract or the eyes, HAdVs can initiate their lytic replication cycle in infected epithelial cells [26]. HAdVs are obligate intracellular pathogens that rely on the host cell replication and transcription machinery to complete their life cycle and produce new virions [27]. Once the adenoviral fiber attaches the host cell surface receptors, an interaction occurs between the conserved RGD motif on the penton base protein and activated cellular integrins, such as αvβ3 and αvβ5 [28]. This interaction facilitates the internalization of the capsid through clathrin-mediated endocytosis [28]. Meanwhile, the fiber proteins remain anchored at the cell surface [28]. Alternative entry pathways, including lipid rafts, caveolin-mediated endocytosis, and macropinocytosis, can also occur [29]. During internalization, partial disassembly of the capsid at the vertex region releases proteins, including the lytic protein VI, which disrupts vesicular membranes, enabling capsid escape into the cytosol [30]. Binding to the nuclear pore complex triggers a cascade of events, including virion priming and uncoating. The ubiquitination of the protein V releases the viral DNA from its associated proteins, allowing for the nuclear import of the adenoviral genome. Viral DNA then interacts with host replication factors, leading to the formation of viral replication compartments where replication occur on an extrachromosomal basis [31]. Following this, new virions assemble within the nucleus, tightly coordinated with viral DNA synthesis to ensure efficient packaging. The infection results in host cell lysis and the release of progeny virions [32].

### 1.2. Immune Evasion and Activation of HAdVs

**Figure 1:**
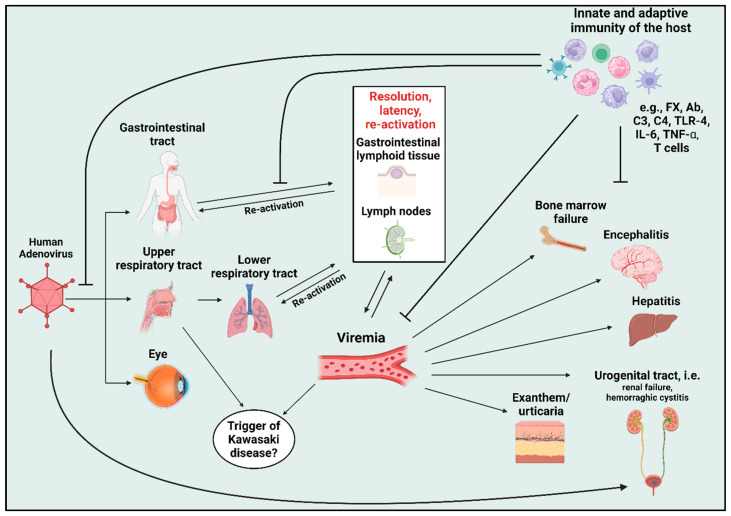
Infection and dissemination of the human adenoviruses (HAdVs) in different organs. The entry route of HAdV is presented by the gastrointestinal and upper respiratory tract, as well as conjunctiva. HAdVs can infect several parts of the lower respiratory tract and lymphoid organs. Viremia may lead to dissemination to other organs, particularly in immunocompromised or critically ill patients. This figure was created using Biorender.com, and the content was adapted from [48]. Abbreviations: FX = Factor X; TLR-4 = toll-like receptor 4; IL = interleukin; TNF-α = tumor necrosis factor alpha. Blunt arrows (┴) indicate inhibition, whereas sharp arrows (→) indicate causal effect.

The formation of new adenoviral progeny enables the spread of the infection in tissues and organs, as well as the invasion of the bloodstream, leading to viremia [33,34]. Once the virus enters the bloodstream, it is covered by soluble factors, including coagulation [35,36,37,38], and neutralizing factors, including natural antibody and complement components (i.e., C3 and C4) [39]. Further immune system activation follows the endocytosis of virions through the interaction of fibers with cellular receptors, and of penton base proteins with integrin, e.g., αvß3, leading to a transcriptional cascade in the host cell [40]. This may involve cytosolic receptors, such as pathogen-associated molecular pattern (PAMP) and transcriptional factors (e.g., NF-kB, IRF3, and IRF7) [41,42,43]. Following this, within 30 min of sequestering adenovirus particles from the blood, this intracellular pathway may lead to the secretion of pro-inflammatory cytokines [44,45], including IL-6, TNF-α, IFNα/β, IL-1α, IL-1β, etc. [46]. The production of such cytokines and chemokines can activate immune cells systemically [47]. This activation may lead to inducing the destruction of HAdVs-containing macrophages through neutrophil-mediated cytotoxicity, stimulate adaptive immune responses, and facilitate the clearance of HAdVs-containing non-phagocytic cells. [47]. Figure 1 illustrates the infectious route after entering the host and inducing immune cell activation and secretion of distinct cytokines and chemokines.

### 1.3. HAdV Diagnosis Techniques and Genotyping

The diagnosis of HAdVs can be performed through different laboratory techniques, including viral culture, (real-time) polymerase chain reaction (PCR), immunofluorescence assays, serological tests, and genotyping. PCR is rapidly available and can detect an infection even at low concentrations, which is furthermore superior to viral cultures and immunofluorescence methods, showing higher accuracy [49,50,51]. For genotyping, sequencing methods, including whole-genome or targeted sequencing of specific genomic regions (e.g., hexon, fiber, or penton base genes) can be also used [52,53,54,55].

### 1.4. Clinical Presentation of HAdVs Infection in Pediatric Risk Population

HAdVs infections are commonly mild (~oligo-symptomatic) in immunocompetent children and thus do not usually lead to hospital admissions, but may be associated with a latency of HAdVs (most frequently observed in children). HAdVs can persist in a latent state in lymphoid organs, including adenoids, tonsils, or Peyer plaques, for several years [56,57,58]. In immunosuppressed pediatric patients, both an acute primary infection and HAdVs reactivation from a latency may occur [10]. This reactivation, due to reduced CD8^+^ T-cell activity and a diminished interferon response, can lead to the virus spreading to different regions of the body and organs, causing multi-organ or systemic infections. [10]. Moreover, a reactivation can be frequently associated with the initiation of or the increase in e.g., immunosuppressive therapy [59], which applies in particular to children undergoing hematopoietic stem transplantation (HSCT). In this patients HAdVs reactivation is associated with a high lethality rate [10,60]. However, neonatal, infant, and adult immunocompetent patients may also present with severe disease manifestations [61,62]. The risk of de novo infection seems to be particularly high at locations where several people regularly meet in a confined space, such as schools, childcare settings, hospitals, and even military barracks [63,64,65,66,67,68,69], possibly due to the surface stability of HAdV and the different infection routes, including contact and droplet infections [23].

Most commonly affected by HAdVs are the respiratory tract and the eyes [70]. In addition, HAdVs may also affect the genital and gastrointestinal tract [71,72]. Infrequently, HAdVs may cause hepatitis [73], carditis [74], hemorrhagic colitis [75,76], pancreatitis [77], nephritis [78], cystitis [79], or meningoencephalitis [70,75,76,80]. Specific HAdV types are associated with infections in specific organ systems, as also presented in Table 1. However, respiratory tract infections are the most common diseases in children caused by HAdVs [76]. Pneumogenic infections due to HAdVs may be a relevant risk factor for developing bronchiolitis obliterans [81,82]. A recently published study revealed a high mortality rate of 12.1% of lower respiratory tract infections among children with congenital heart disease caused by HAdVs [83].

In detail, respiratory tract infections are commonly caused by HAdV-C1, HAdV-C2, HAdV-C5, HAdV C6, as well as HAdV-B3, HAdV-B7, HAdV-B14, HAdV-B21, HAdV-B55, HAdV-B66, and HAdV-E4 [85], with types of species C rather restricted to the upper respiratory tract, whereas types of species B and species E have a tendency to also infect the lower respiratory tract. In particular, infections caused by HAdV-B3 or B7 have been associated with higher hospitalization rates compared to other HAdV types. [86]. Furthermore, respiratory tract infections in younger adults, including U.S. military recruits, have shown more severe clinical courses when caused by HAdV-E4 or HAdV-B7. Thus, the only vaccine, which is currently reserved for this patient group, is specifically protecting against these HAdV types [87]. In addition, a more severe outcome of HAdV-B7 infection has been reported [88], associated with increased morbidity and mortality rates [89]. The recombinant type of HAdV-B66 (originating from types 3 and 7, primarily labeled as type 7h) may cause severe lower respiratory tract infections, especially in South America [90,91]. Furthermore, HAdV-B14a, which is a slightly modified subtype of HAdV-B14, caused large outbreaks of lower respiratory tract infections and was considered a re-emergent virus in the USA [92]. In China, however, HAdV-B55 is a relevant pathogen leading to community-acquired pneumonia [93,94]. In fact, HAdV-B55 is a recombinant virus with most of its genome originating from HAdV-B14 [95]. Its neutralization epitope originates from HAdV-B11 and thus achieves an immune escape and may lead to mislabeling in HAdV classification protocols. A further, likely re-emergent type of virus that is associated with a lower respiratory tract infection is HAdV-B21a [85,96]. The probability of the requirement of hospitalization during a respiratory HAdV-infection is positively correlated with immunosuppression [97]. A high fatality rate for a disseminated HAdV-infection in patients with HSCT was reported in multiple studies [3,98,99,100,101,102,103,104]. However, the majority of HAdV-infections in HSCT patients originate from the reactivation of latent infections [9,10]. Therefore, children with HSCT are at an increased risk for disseminated infections compared to the adult population undergoing HSCT [99,105]. Due to an increase in mortality and morbidity, HAdV-infections have a significant relevance in this patient population. Here, HAdV types of species C (HAdV-C1, HAdV-C2, HAdV-C5) and species A (HAdV-A31) are the most prevalent pathogens detected in severe courses in those HSCT patients [98].

## 2. Methods

HAdVs dissemination differs according season, region, as well as genotype [75,106], and a higher prevalence rate mostly correlates with cold weather [107]. Considering these serious regional differences, a systematic approach is required for a comprehensive overview of clinically relevant HAdV types. To summarize the emerging HAdV types and point out the clinical relevance of these types, we thus performed a literature search using PubMed/MEDLINE, Scopus, and Web of Science on 8 March 2024, aiming for an overview of newly diagnosed and reported HAdV types in previous years. We updated our search results again on 26 October 2024, and the study was conducted in accordance with the PRISMA [108] and Joanna Briggs Institute (JBI) methodology for systematic review guidelines [109]. A protocol was registered for this systematic review on PROSPERO [110], with the following registration number: CRD42024606505 (https://www.crd.york.ac.uk/PROSPERO/view/CRD42024606505, first submission to PROSPERO on 26 October 2024). An ethics approval was not required.

### 2.1. Search Strategy

A structured three-step search strategy was used to locate published and unpublished studies for inclusion in this systematic review. A preliminary search of MEDLINE was performed to determine index terms. A full search strategy was then developed, with databases MEDLINE, Scopus, and Web of Science searched. Keywords related to “HAdV” and “children” were used and combined with BOOLEAN operators “AND” and “OR”. The search was limited to prospective studies published in English or German, between 1 January 2023 and 25 October 2024. We used the search term “adenovirus AND (children OR child OR infant OR childhood OR pediatric OR paediatric)” in all three databases. All identified studies during the search term were transferred to Excel. Titles and abstracts of all studies were subsequently screened by different independent reviewers (J.S.W., C.K., and L.S.). Full-text studies that met the inclusion criteria were obtained and reviewed by different independent reviewers (J.S.W., C.K., and L.S.). Conflicts were discussed and decided by a fourth independent reviewer (M.A.). The reference lists of the final selected studies were assessed for the possibility of additional sources.

### 2.2. Selection Criteria

**Figure 2:**
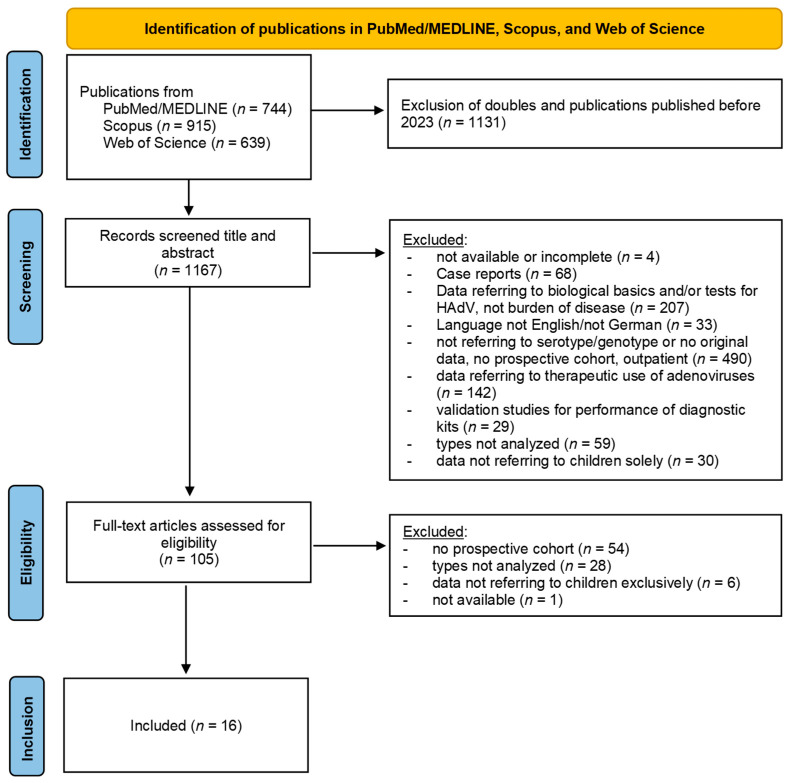
Flow diagram according to PRISMA 2020 recommendations according to Page et al. (2021) [108].

Studies were included if they were published between 1 January 2023 and our search date. To be eligible for inclusion, studies had to report on the specific genotypes that led to HAdV-infection and hospitalization. Reports on outpatient settings were not included. Due to our aim to obtain the most comprehensive overview for currently circulating HAdV types and infections in children, only prospective studies were included. Studies including both adults and children were considered if data for adults were reported separately. In addition, studies were excluded when there were duplicate publications, did not specify the age ranges, or did not correspond to infections caused by HAdV. We excluded articles published in neither English nor German, that did not contain data on HAdV genotypes, that did not report original data, referred to biological and/or therapeutic principals instead of disease burden, or tested laboratory techniques or the use of adenoviral vectors, as well as case reports. Our selection process is demonstrated in the following PRISMA Flowchart according to Page et al. [108] (Figure 2).

After excluding those duplicate articles, a total of 1167 articles were considered for further analysis. These articles were screened, sorted, and assessed regarding reported HAdV types. After the initial screening, a total number of 105 articles were further assessed for eligibility. Of these 105 articles, 16 provided detailed information on prospective studies performing complete genotyping of reported HAdV-infections in children. The characteristics of each included study are detailed in Table 2. The full list of articles screened is provided in Supplementary Table S1.

### 2.3. Comprehensive Analysis and Evaluation

The selected studies were critically appraised by two independent reviewers using the JBI critical appraisal tool [127] (Table 3). This tool evaluates several dimensions of study quality, including participant selection, outcome measurement, and study comparability. Each criterion received a score based on the following scale: yes = 2 (green), no = 0 (red), and unclear = 1 (yellow). It is important to note that the scoring process involved some degree of subjective judgment, which may influence the final evaluation, and that missing information may lead to a lower score without necessarily representing a lack of methodological quality, but rather a lack of description. Any discrepancies in scoring were discussed and addressed with the involvement of an additional team member. All studies, regardless of their methodological quality, were included in the review. The methodological quality of the included studies varied, with scores ranging from 8 to 18. Detailed descriptions of study subjects and settings were provided in all except two studies. Although there were no studies excluded, it is thus important to consider the risk of bias when evaluating the results.

### 2.4. Data Extraction and Synthesis

The data extraction was completed by two reviewers and checked by a third author. The extracted data included author, year of publication, country, total sample size, and identified HAdV types. The instrument used for data extraction was a purpose-designed Excel spreadsheet developed by the authors, provided in the Supplementary Material (Supplementary Table S2). The data on HAdV types from the included studies were calculated additionally. To ensure the accuracy of the data entry, this step was conducted simultaneously by two different reviewers and double cross-checked.

## 3. Results of the Systematic Research on Emerging HAdV Types

Our systematic review identified 16 studies: six articles were published from China [111,115,117,121,125,126]; *n* = 2 each from Tunisia [112,122], India [118,119], and Iran [114,120]; as well as *n* = 1 from each Vietnam [113], Thailand [116], Slovenia [123], and Uganda [124]. The risk of bias is presented above. Most of the studies had only a small risk of bias according to the bias assessment, and four had a moderate risk of bias according to the JBI assessment [112,114,120,122].

Four articles reported on more than one clinical symptom complex. Seven studies reported on patients with gastrointestinal symptoms, nine on respiratory tract infections, one reported on measles-like illness, and three reported on neurological symptoms. When summarized, the 16 articles provided information on 911 successfully genotyped HAdV samples, with fully detailed information regarding genotyping. The frequency of HAdV species is summarized in Table 4.

### 3.1. Respiratory Tract Infections

Respiratory tract infections were prevalently reported on types of HAdV-B3 and HAdV-B7 (and combinations of both, including the recombinant HAdV-B66). Two studies linked HAdV-B3 and -B7 to the causation of local outbreaks of respiratory tract infections, particularly in children under five, where these strains were associated with more severe conditions like lower respiratory infections [113,118]. Similarly, concomitant co-infections were associated with more severe respiratory diseases [113]. An increased disease severity associated with co-infections was also observed during the previous COVID-19 pandemic, though other genotypes were more prevalent in those cases (HAdV-C1, -C2, and -C6) [115].

### 3.2. Gastrointestinal Tract Infections

Acute gastroenteritis (AGE) was caused by HAdV-F40 and HAdV-F41 and was associated with a higher prevalence rate in children under the age of five [111,112,114] in Yunnan, China [111] (years 2015 to 2021), as well as in Tunisia (2014 to 2016) [112] and in Iran (2021 to 2022) [114]. Moreover, HAdV-F41 and HAdV-C2 [114,122] were frequently detected, reflecting the global predominance of these serotypes in pediatric AGE [128,129,130]: Following a decline in HAdVs-detection during COVID-19 restrictions, after the pandemic, there was an atypical increase in outpatient infections with HAdV-F41 in Shanghai [131], assuming an indirect correlation and influence of pandemic measures on HAdV epidemiology [132]. This study is in accordance with the post-pandemic high incidence rate of HAdV-F41 infections reported in the UK [133], which was not identified by the systematic search because it was published in 2022 in the context of investigations on hepatitis of unknown etiology. Later publications rather identified Adeno-Associated Virus 2 co-infections with HAdV-F41 as the most likely etiology of this hepatitis outbreak [134,135].

HAdV-C2 was reported in a relevant proportion (105 out of 911 total) [111,113,114,115,116,122,123] and correlating to respiratory and gastrointestinal infections [115,122]. Of note, HAdV-C2 is the only type reported from every country in which the included studies were conducted. This highlights its worldwide endemic distribution and significance in disease burden.

### 3.3. Neurological Infections

While the prevalence of HAdV-F40 and HAdV-F41 in patients with AGE, and HAdV-B3 and B7 in respiratory tract infections are well described, HAdVs involvement in cerebral infections is insufficiently known. In detail, only one of the reported studies conducted RNA and DNA sequencing of viral pathogens in meningitis and encephalitis with HAdV detection in cerebrospinal fluid and serum, where the number of the genotyped cases reported were small (*n* = 8) for HAdV-B3 and -B7 [117]. A further study revealed the association between HAdV and patients with febrile seizures [123]. Another study linked HAdV to measles-like illnesses, but it only reported a lower number of HAdV-positive infections (*n* = 4) [124].

## 4. Discussion

This systematic research included results about HAdVs typing in immunocompetent and immunosuppressed pediatric patients. A precise statement about the ratio of the immunocompetence of the respective patients cannot be performed. All the reported studies focused on the HAdV types rather than the clinical details of the patient population. However, it is well known that HAdV types observed in immunosuppressed patients rather originate from the reactivations of latent HAdV-DNA [136,137]. As HAdV-DNA latency is a consequence of endemic infections of children with HAdV-C types, these results rather demonstrate the continuous endemic significance of HAdV-C2 but do not correlate with the re-emergence of HAdV types. However, certain clinically significant manifestations, such as meningoencephalitis, disseminated cutaneous involvement, and bone marrow failure, are rarely observed in immunocompetent children. A reduction in cellular immunity, specifically T-cell-mediated immunity, is the most significant risk factor for a dysregulated and inefficient immune response against HAdVs. In immunosuppressed children, the reactivation of a latent HAdV-infection is a distinct pathomechanism that can lead to severe disease progression. Both congenital immunodeficiency (such as severe combined immunodeficiency syndrome, agammaglobulinemia, immunoglobulin A deficiency, hyper-immunoglobulin M syndrome) and acquired immunodeficiency (e.g., acquired immunodeficiency syndrome, severe malnutrition or immunosuppressive therapy) are common risk factors for severe HAdV-infections [138]. HSCT in children has become increasingly common, with a growing range of indications and improved outcomes. Consequently, this is also associated with an increased clinical relevance of infections and HAdVs reactivations in immunocompromised children. Importantly, the study reported here with the most severe courses of the disease (14 out of 45 patients deceased) due to HAdV-B7 did not include any patients with immunosuppression [89]. This severe outcome in non-immunosuppressed patients underscores the requirement to explore host–pathogen interactions beyond solely immune suppression. In patients with rapid and severe disease progression, the effect of viral load, “cytokine storm”, and co-infections, as well as the neurotropic potential of certain strains require further investigation. We observed two studies correlating HAdV-B7 to neurological symptoms in children and another study reporting those patients to be immunocompetent, without detailing in one further report. Our results presented here might be influenced through the origin of the included studies. They were performed in China or South Asia, which potentially do not represent the populations in Europe, the USA, or Africa. In addition, data may also be biased as many of the papers are related to outbreaks of HAdV-infections and therefore may not represent the true epidemiological burden of disease due to HAdV. The included papers covered different clinical manifestations and age groups related to all types of HAdV variations. However, we could not make clear comparisons of HAdV types with those from other cities or identify patterns in certain populations, as these data were not available in the literature despite our systematic search approach. Thus, understanding the mechanisms driving those severe manifestations still remains a critical field of further study. More observations and studies need to be conducted and published to increase awareness of HAdV-infections in hospitalized children.

## 5. Conclusions

With this article, our primary aim was to provide an overview of the recently reported HAdV genotypes and to increase awareness of HAdVs in hospitalized children. The results of our systematic literature search may be compromised by the typing procedures used in the studies, especially if completed genomic sequencing and analysis of genomic sequences for recombinant phylogeny were not conducted according to the state of the art and only single genes were sequenced. This may lead to the primary description of outbreaks with novel HAdV types (as mentioned in the introduction section regarding HAdV-B55 and -B66 types) under a misclassified typing result for an old type (e.g., HAdV-B7 or HAdV-B11). At this level, we may not rule out biases due to the restricted assessed timeframe for our literature search. Moreover, the scope of our search was focused on articles published recently but also included datasets from older prospective studies (e.g., a study from Slovenia performed during the years 2009 to 2011 [123]). Moreover, the evaluation did not include an assessment on the correlation between specific genotypes and their associated clinical manifestations or disease severity. However, this review indicates that the diversity of clinically relevant circulating strains in children may be significantly greater than previously considered. Thus, due to the significant influence of regional and meteorological phenomena on the prevalence of clustered infections, special attention to specific preventive measures and the continuous surveillance of circulating HAdV types is highly relevant.

## Figures and Tables

**Table 1:** Commonly involved HAdV types and clinical symptoms.

Symptoms	Types
Conjunctivitis	B3, E4, B7, D8 *, B14, D19, B21, D37 *, D53 *, B55, D64 *
Pharyngitis	C1, C2, B3, C5, C6, B7, B14
Respiratory tract (more frequently restricted to the upper respiratory tract)	C1, C2, B3, C5, C6, B14, B21
Respiratory tract (more frequently also affecting the lower respiratory tract)	E4, B7, B14a, B21a, B55, B66
Acute Gastroenteritis (* epidemic in toddlers and infants)	C1, C2, C5, C6, A12, A18, A31, F40 *, F41 *
Acute Cystitis	B11, B34, B35
Infections (or reactivations) in immunocompromised patients	C1, C2, C5, C6, A31

Adapted and supplemented according to Heim A. (2020) [84]. * HAdV types causing symptomatic Keratoconjunctivitis.

**Table 2:** Studies included in our systematic review.

Study	Place	Time Frame the Study Was Conducted	Main Reported Symptoms
Detection and complete genome sequence analysis of human adenovirus in children with acute diarrhea in Yunnan, China, 2015–2021 [111].	Yunnan province (China)	2015–2021	Gastroenteritis
Molecular analysis of adenovirus strains responsible for gastroenteritis in children, under five, in Tunisia [112].	Tunisia	2014–2016	Gastroenteritis
Molecular subtypes of Adenovirus-associated acute respiratory infection outbreak in children in Northern Vietnam and risk factors of more severe cases [113].	Northern Vietnam	2022	RTI; Gastroenteritis
Molecular prevalence and genotype distribution of human adenovirus in Iranian children with gastroenteritis [114].	Iran	2021–2022	Gastroenteritis
Molecular epidemiology and phylogenetic analyses of human adenovirus in pediatric patients with acute respiratory infections from Hangzhou during COVID-19 pandemic [115].	Hangzhou (China)	2020–2021	RTI
Diverse genotypes of human enteric and non-enteric adenoviruses circulating in children hospitalized with acute gastroenteritis in Thailand, from 2018 to 2021 [116].	Thailand	2018–2021	Gastroenteritis
RNA-sequencing-based detection of human viral pathogens in cerebrospinal fluid and serum samples from children with meningitis and encephalitis [117].	Hunan province (China)	2020	CNS-infection
Upsurge in hospitalization of pediatric patients with severe acute respiratory infections in Kolkata and surrounding districts caused by recombinant human respiratory adenovirus type B 7/3 [118].	India	2022–2023	RTI
Enteric and non-enteric adenoviruses in children with acute gastroenteritis in Western India [119].	India	2013–2016	Gastroenteritis
Phylogenetic characterization of rhinovirus and adenovirus in hospitalized children aged ≤ 18 years with severe acute respiratory infection in Iran [120].	Iran	2018–2019	RTI
Pneumonia in children during the 2019 outbreak in Xiamen, China [121].	Xiamen (China)	2019	RTI
Unexpected predominance of human adenovirus F41 in children suffering from acute respiratory infection in Tunisia [122].	Tunisia	2018–2019	RTI
Molecular typing of mastadenoviruses in simultaneously collected nasopharyngeal swabs and stool samples from children hospitalized for acute bronchiolitis, acute gastroenteritis, and febrile seizures [123].	Slovenia	2009–2011	RTI; Gastroenteritis; Neurological symptoms
Viruses associated with measles-like illnesses in Uganda [124].	Uganda	2010–2019	Measles-like symptoms
Genotypes and phylogenetic analysis of human adenovirus in hospitalized pneumonia and influenza-like illness patients in Jiangsu Province, China (2013–2021) [125].	Jiangsu Province (China)	2013–2021	RTI; influenza-like illness (ILI)
Genetic characterization of pediatric SARI-associated human adenoviruses in eight Chinese provinces during 2017–2021 [126].	China	2017–2021	RTI

Abbreviations: RTI = respiratory tract infection; CNS = central nervous system, ILI = Influenza-like illness.

**Table 3:** Critical appraisal results of eligible studies.

	1. Was the Sample Frame Appropriate to Address the Target Population?	2. Were Study Participants Sampled in an Appropriate Way?	3. Was the Sample Size Adequate?	4. Were the Study Subjects and the Setting Described in Detail?	5. Was the Data Analysis Conducted with Sufficient Coverage of the Identified Sample?	6. Were Valid Methods Used for the Identification of the Condition?	7. Was the Condition Measured in a Standard, Reliable Way for All Participants?	8. Was There Appropriate Statistical Analysis?	9. Was the Response Rate Adequate, and If Not, Was the Low Response Rate Managed Appropriately?
Cao et al. (2024) [111]	**✔**	**✔**	**✔**	**✔**	**✔**	**✔**	**✔**	**✔**	**✔**
Bouazizi et al. (2024) [112]	**✔**	**✔**	**?**	**✔**	**?**	**✔**	**✔**	**-**	**?**
Nguyen et al. (2023) [113]	**✔**	**✔**	**?**	**✔**	**✔**	**✔**	**✔**	**✔**	**✔**
Kadhim Jwaziri et al. (2023) [114]	**?**	**?**	**?**	**-**	**-**	**✔**	**?**	**-**	**✔**
Huang et al. (2023) [115]	**✔**	**✔**	**✔**	**✔**	**✔**	**✔**	**?**	**✔**	**✔**
Yodmeeklin et al. (2023) [116]	**✔**	**✔**	**✔**	**✔**	**✔**	**✔**	**✔**	**✔**	**✔**
Fan et al. (2023) [117]	**✔**	**✔**	**✔**	**✔**	**✔**	**✔**	**✔**	**✔**	**✔**
Majumdar et al. (2023) [118]	**✔**	**✔**	**✔**	**✔**	**✔**	**✔**	**?**	**✔**	**✔**
Joshi et al. (2023) [119]	**✔**	**✔**	**✔**	**✔**	**✔**	**✔**	**✔**	**✔**	**✔**
Abbasi et al. (2023) [120]	**✔**	**✔**	**✔**	**-**	**✔**	**✔**	**✔**	**-**	**✔**
Zhang et al. (2023) [121]	**✔**	**✔**	**✔**	**✔**	**✔**	**✔**	**✔**	**✔**	**✔**
Bouazizi et al. (2023) [122]	**✔**	**✔**	**-**	**✔**	**-**	**✔**	**✔**	**?**	**?**
Biškup et al. (2023) [123]	**✔**	**✔**	**✔**	**✔**	**✔**	**✔**	**✔**	**✔**	**✔**
Namuwulya et al. (2024) [124]	**✔**	**✔**	**✔**	**✔**	**-**	**✔**	**?**	**✔**	**✔**
Wang et al. (2024) [125]	**✔**	**✔**	**✔**	**✔**	**✔**	**✔**	**✔**	**✔**	**✔**
Cai et al. (2024) [126]	**✔**	**✔**	**✔**	**✔**	**✔**	**✔**	**✔**	**✔**	**✔**

The background colors and punctuation marks indicate the assumed bias, green and checkmark indicate no or low risk of bias, yellow and question mark indicate uncertain risk of bias and red and hyphen indicate risk of bias.

**Table 4:** Frequency of reported HAdV species in analyzed samples (*n* = 2888).

HAdV Species (*n* = Samples Reported)	Type (*n* = Samples Reported)
HAdV-A (*n* = 16)	A12 (*n* = 7)
	A18 (*n* = 3)
	A31 (*n* = 6)
HAdV-B (*n* = 463)	B3 (*n* = 225)
	B7 (*n* = 193)
	recombinant B3/B7 (*n* = 40)
	B11 (*n* = 1)
	B16 (*n* = 2)
	B55 (*n* = 2)
HAdV-C (*n* = 291)	C1 (*n* = 83)
	C2 (*n* = 105)
	C5 (*n* = 38)
	C6 (*n* = 50)
	C57 (*n* = 3)
	C89 (*n* = 1)
	C108 (*n* = 11)
HAdV-D (*n* = 2)	D23 (*n* = 1)
	D69 (*n* = 1)
HAdV-E (*n* = 11)	E4 (*n* = 11)
HAdV-F (*n* = 128)	F40 (*n* = 30)
	F41 (*n* = 98)
Not specified	NN (*n* = 1977)

## Supplementary Materials

The following supporting information can be downloaded at: https://www.mdpi.com/article/10.3390/v17070914/s1, Table S1: Full list of screened articles for studies reporting HAdV genotyping in pediatric infections; Table S2: Purpose-Designed Excel spreadsheet used for data extraction.
